# Thrombotic Microangiopathy Secondary to Intravenous Abuse of Opana® ER

**DOI:** 10.1155/2017/1623907

**Published:** 2017-05-18

**Authors:** Kamia Thakur, Vaibhav Agrawal, Ashley Kass, Lauren M. Dimarino, R. Patrick Dorion, Joseph Vadakara

**Affiliations:** Geisinger Medical Center, Danville, PA, USA

## Abstract

Opana ER (oxymorphone) is an opioid drug available throughout the United States, and intravenous abuse of the crushed oral formulation has been associated with drug-induced thrombotic microangiopathy. In this abstract, we describe two young patients who lived together and used Opana ER intravenously. Both presented with microangiopathic hemolytic anemia that mimicked thrombotic thrombocytopenic purpura (TTP). Treating this condition poses a clinical challenge, as it is difficult to distinguish it from TTP. The role for plasma exchange is not clear but can be used while awaiting the results of the ADAMTS-13 activity, but ultimately supportive care with drug discontinuation is the recommended therapy of choice. Patients should be counseled against Opana ER's intravenous use, and users should be offered drug rehabilitation therapy.

## 1. Introduction

We present a case series of two patients who developed thrombotic thrombocytopenic purpura (TTP) like drug-induced thrombotic microangiopathy (DITMA) following the intravenous use of Opana ER. This case series brings awareness to the growing concern of developing DITMA in those who inject Opana ER.

Opana ER or oxymorphone is a semisynthetic opioid introduced in 1955 by Endo Pharmaceuticals [1]. The FDA originally approved Opana ER in 2006, and this formulation of Opana ER allowed it to be crushed to a fine powder and snorted. In 2012, a crush resistant formulation was developed by Endo Pharmaceuticals to help curtail abuse of the drug. With this formulation, the drug could no longer be crushed and snorted, but this did not prevent it from being used intravenously [2, 3].

## 2. Case Presentation

**Figure 1:**
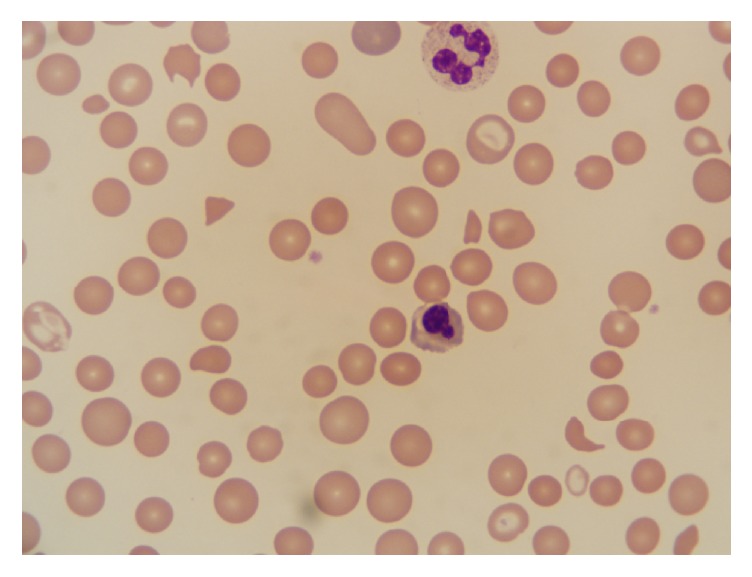
Schistocytes and thrombocytopenia on peripheral blood smear of Case A.

A 26-year-old female (Case A) with a past medical history of polysubstance abuse presented with complaints of generalized weakness and progressively worsening left upper quadrant abdominal pain. She also complained of a visual field defect in her right eye. On admission, she was found to be tachypneic, tachycardic, and afebrile. Laboratory studies showed hemoglobin of 5.1 g/dL with schistocytes and thrombocytopenia on peripheral blood smear (Figure 1), platelet count of 62 K/uL, BUN of 22 mg/dL, and creatinine of 1 mg/dL (baseline 0.6 mg/dL). LDH and haptoglobin were 584 U/L and <10 mg/dL, respectively, and direct Coombs' test was negative. PTT was 32 seconds (21–38 seconds), and soluble fibrin monomers were absent in serum. A presumptive diagnosis of TTP was made, and plasma exchange was initiated after an ADAMTS-13 level was drawn. Further workup revealed a positive HCV antibody test with undetectable HCV RNA levels. A fundoscopic exam showed cotton wool spots. The patient admitted to abusing Opana ER intravenously every day for the past couple of years, and this was confirmed with urine drug testing which was positive for oxymorphone. Plasmapheresis was discontinued after ADAMTS-13 levels obtained at admission returned at 69%, and Opana ER was considered the causal agent. With supportive care, she made a complete recovery including improvement of her vision.

Case A was readmitted to the hospital a few months later with septic arthritis. Laboratory studies were consistent with microangiopathic hemolytic anemia with thrombocytopenia similar to her prior admission. Her hemoglobin at presentation was 4.8 gm/dL with schistocytes and thrombocytopenia with platelet count of 62 K/uL, BUN of 22 mg/dL, and creatinine of 2 mg/dL (baseline 0.6 mg/dL). LDH and haptoglobin were 898 U/L and <10 mg/dL, respectively. Her PT was 14.3 seconds (normal limit 11.4–14.6 seconds) but PTT and fibrin monomers were not obtained to rule out disseminated intravascular coagulation (DIC) on account of her admitted use of intravenous Opana ER for several days before this hospitalization. The use was confirmed by urine drug screen, which was positive for oxymorphone. Her septic arthritis was managed with prompt surgical debridement and antibiotics, and her hematologic profile recovered without plasmapheresis. The patient recovered fully with this supportive care, and she was counseled against continued Opana ER abuse.

**Figure 2:**
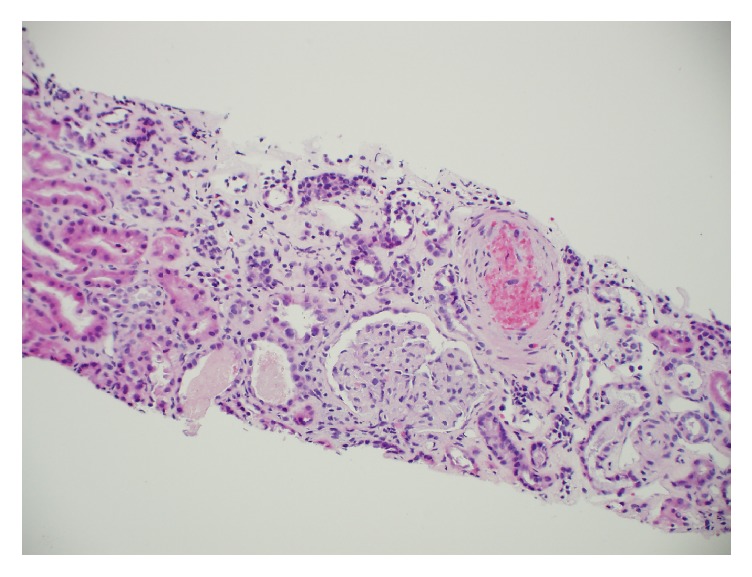
Renal biopsy of Case B showed evidence of a thrombotic microangiopathic process.

The second case (Case B), a 22-year-old female, was Case A's roommate. She too had an extensive history of intravenous drug use. She was admitted to the hospital a month before Case A with respiratory failure, altered mental status, and renal failure of unclear etiology. Laboratory studies showed hemoglobin of 7.5 g/dL, platelet count of 129 K/uL, BUN 137 mg/dL, and serum creatinine of 12.9 mg/dL. A peripheral blood smear revealed schistocytes and thrombocytopenia. LDH was 2027 U/L, and haptoglobin was <10 mg/dL. A presumptive diagnosis of TTP was made. ADAMTS-13 levels were drawn, and plasma exchange was initiated. She was positive for hepatitis C antibody, and HCV quantitative RNA was 2056 IU/mL. She was not on any therapy for hepatitis C. Other autoimmune and infectious workups were unremarkable. Due to rapidly declining renal function, a renal biopsy was obtained. The biopsy showed evidence of a thrombotic microangiopathic process (Figure 2). Plasmapheresis was discontinued when ADAMTS-13 levels obtained at admission returned at 33%. With intensive care and supportive measures, her hematologic profile improved. However, she remained dialysis dependent. Based on her clinical picture she was thought to have atypical hemolytic uremic syndrome, and plans were made to start eculizumab. The true cause of her condition remained unclear until her friend (Case A) was admitted with similar issues. At this time, she too admitted to abusing Opana ER intravenously, which was confirmed by a positive urine test. With intensive care and supportive measures, her hematologic profile improved. However, she remained dialysis dependent.

## 3. Discussion

Thrombotic microangiopathy (TMA) has been previously reported with several medications, including vascular endothelial growth factor inhibitors, calcineurin inhibitors, interferon, gemcitabine, mitomycin-C, clopidogrel, and quinine.

The cause of TMA in patients who abuse Opana ER is not clearly understood. It is thought to be either an immunologically mediated response or a chronic dose-dependent toxic drug effect [4]. The reformulated version of Opana ER contains polyethylene oxide (PEO) and polyethylene glycol (PEG) [5]. TMA with intravenous abuse of Opana ER may be due to the PEO. Prior studies in rats have implicated PEO as a causative substance by demonstrating dose-dependent hemolytic anemia and thrombocytopenia following IV and intraperitoneal injection of a high molecular weight PEO obtained by concentrating Opana ER tablets [6]. Hunt et al. dosed guinea pigs with PEO and found that this can trigger hematologic responses resembling elements of TTP, including intravascular hemolysis and renal injury/failure [7]. Similarly, Nataatmadja and Divi report the incidence of TMA in a patient intravenously using two different opioids, with both the same reformulated PEO coating and the use of only mechanical methods to prepare the medication for injection [8]. The use of IV oxymorphone alone is not known to cause TMA, suggesting that the causative agent is a substance other than oxymorphone in the tablets. This obscurity of the causative agent from the inert mixture of oxymorphone, PEO, and PEG differentiates Opana ER induced TMA from other DITMA.

There is growing evidence that viruses may play an important role as triggers in the pathogenesis of thrombotic microangiopathies. Multiple viruses have the capacity to induce endothelial dysfunction, which is implicated in the development of TMA [9]. Both Cases A and B had HCV antibodies, but only Case B had a viral load at presentation. Similarly, the initial CDC report released in 2013 which identified the series of 15 cases of TTP-like illness secondary to Opana ER in Tennessee noted that 12 of the 15 patients had chronic hepatitis C or had positive anti-HCV antibodies. Anticardiolipin antibodies have been linked with chronic HCV infection, and ADAMTS-13 inhibition has been implicated in the development of HCV-related TMA [10].

The clinical presentation of DITMA due to intravenous Opana ER use is similar to atypical HUS or TTP and includes microangiopathic hemolytic anemia, thrombocytopenia, fever, renal insufficiency, neurological symptoms, and thrombotic microangiopathy. Most cases have the typical presentation, but the diagnosis is challenging, as there are no specific laboratory tests available that help to make an early diagnosis. ADAMTS-13 activity is usually normal to moderately decreased and can be drawn to differentiate it from TTP [11–13]. Cases A and B had normal and moderately decreased ADAMTS-13 levels, respectively, before initiating plasmapheresis.

Whether only supportive care is helpful, or if there is utility in plasma exchange, is yet to be determined. In a previously reported case series of 15 patients with Opana ER associated TMA plasma exchange was not done, and patients were managed supportively after drug cessation, with successful outcomes [14]. Given the diagnostic challenge of clinically differentiating Opana ER related TMA from TTP and in situations where there is not any evident history of IV drug abuse, therapeutic plasma exchange should not be delayed to either observe the clinical course or await ADAMTS-13 results. Case A received plasmapheresis on her first presentation, but the hematological profile improved with only supportive care at the second presentation. Discontinuation of Opana ER use reverses the pathologic process. Long-term outcomes in patients treated with or without plasma exchange are unknown due to lack of follow-ups in this population.

Physicians and health care providers managing thrombotic microangiopathy should consider intravenous Opana ER use as a possible cause. Urine testing for oxymorphone can be used to confirm the history or when the patient is unsure of what they have been using. A lack of familiarity, as was seen in the management of Case B, can lead to diagnostic errors. Patients should also be counseled against the intravenous use of Opana ER and should be offered rehabilitation programs. Reuse of Opana ER after an episode of DITMA may cause a relapse as was seen in Case A.
